# The Syphilisation of Children

**Published:** 1857-02

**Authors:** B. W. Boeck

**Affiliations:** Professor de Medicine, á l’Université de Christiana


### ARTICLE VI.

From the Archives Generales.

*The Syphilisation of Children.* By B. W. Boeck, Professor de Medicine, à l'Université de Christiana. Translated by J. J. West, M. D., Dem. Anatomy, Savannah Medical College.

Syphilisation, one of the most beautiful discoveries of our time, has found none but opponents. The Academy of Medicine of Paris, has battered in the breach this new theory, without having sufficient elements of decision. A great number of physicians have followed blindly its judgment; and, having once taken sides, they have rejected the doctrine as ridiculous, without calling in the aid of experience. However, the therapeutic agents, which are employed to combat Syphilis, are neither sufficiently infallible, nor so exempt from inconveniences that we should not encourage honest attempts, however paradoxical they may appear at first sight. If observers had desired to follow serious experiments, they would have acquired a conviction of the reality of Syphilisation and its good effects.

Since the day the method was first known to me, I have undertaken a series of uninterrupted researches: my first trials, by the concurrence of different circumstances, date only from the year 1852. The results which I obtained and which I have already noticed in former publications, can be resúmed thus:

1st. Inoculation of the syphilitic virus, sufficiently prolonged, determines an absolute immunity.

2d. The syphilitic manifestations which are produced at the commencement of syphilisation disappear, however long the inoculations may be continued.

3d. The general health is in no way altered by syphilisation; on the contrary, the patient feels himself better than before the treatment.

These conclusions, arrived at since 1854, have served me as a point of departure for ulterior researches. Although the principal end of this memoir is, as indicated by the title, to treat of syphilisation, specially upon infants, I think it useful to expose, in a few words, the observations, which, in the last two years, I have instituted upon adults. Besides, that some explanations may not be useless to French readers, they will assist to judge of the actual state of individuals syphilised, and of their liability to a recurrence of the disease.

I have treated in the last two years 63 persons, of whom 36 had never been submitted to any medication, and 27 had followed a mercurial treatment. It is superfluous, I hope, to state, that I only employ syphilisation for persons affected by constitutional syphilis; and, consequently, I introduce into the organism only the morbid elements it has already received. I have always been the declared adversary of prophylactic syphilisation. It is, I think, an absurd idea, to pretend to preserve men from a malady which they contract only by an act of their own will. Nothing is changed in the mode of syphilisation I have employed thus far.

I take the matter of a primitive chancre, and inoculate it into the patient by the arms and thighs; most frequently, I make three upon each arm and thigh. However, I have, in some cases, inoculated upon the sides of the chest, instead of the place of election adopted by Sperino, so that the cicatrices may be less apparent. The choice of the part of the body that one inoculates is not indifferent. Upon the arms and breast the inoculations are smaller, and of shorter duration than upon the thighs; and save rare exceptions, it is there I inoculate by preference. I employ, ordinarily, the matter of previous inoculations, for those that are practiced subsequently. If the last inoculations have not taken well, I do not hesitate to have recourse to the pus of anterior ones. In some cases I have served myself, only of ulcerations, dating from the commencement of the syphilisation, inasmuch as if they furnish an inoculable pus, supposing that the matter was active, the treatment should be shorter. I have not, as yet, experimented enough to know whether or not this supposition is well founded; but the question is important, and merits that I return to it later in detail. It often happens in our country, that, when one takes the virus to inoculate it upon persons who have contracted their chancres abroad, the ulcerations are extensive, and become phagedenic; but there is no reason to be alarmed at this; on the contrary, it must be persevered in. Even should the series of inoculation be interrupted, the phagedenism makes a progress that would be infallibly arrested by the syphilisation. If we employ a matter modified by transmission through different individuals, and by successive inoculations, the ulcerations never assume the phagedenic character, and it is thus, that in the last few months, not having at my disposal the fresh virus, I have not observed a single phagedenic ulcer upon those persons I have inoculated. I have concluded from this, that it would be well to employ always in the commencement of syphilisation, a matter which has passed through several inoculations, and to continue this until the syphilitic susceptibility has been somewhat enfeebled. Is this matter less energetic, less syphilising? Should the syphilisation be practiced for a longer time? These are questions difficult of solution, yet I am inclined towards the negative.

When one matter ceases to give a satisfactory result, I have had recourse to another; and thus on until I could find inoculable pus. In inoculating in this way, each patient has had more than a hundred chancres, and a large number have passed that figure considerably. Let us hasten to add, that among these hundred chancres, can be counted rarely more than twenty or thirty of any extent, or which leave evident cicatrices; the others reduce themselves to simple excoriations. It is sufficiently probable, that these last inoculations, which do not produce characteristic ulcers, are not indispensable to the success of the treatment. However, I have always insisted upon this practice; either to study well the action of the virus, or because it is necessary when a method is new to multiply guaranties. It is equally probable, that one would be able to dispense with a repetition of twelve inoculations every three days, and give a longer space of time between them. Perhaps a small number of inoculations might suffice for thorough syphilisation, but the treatment, as I have employed it, has succeeded, and I have feared, lest in changing it, the results might be injured.

Two or three months are generally necessary to render the last inoculations entirely negative. After that time the immunity is perfect--should one inoculate the virus of syphilis or distilled water, the effect would be the same—nothing. This law suffers no exceptions, and the incredulous may convince themselves with facility.

The mode of operating once exposed, I return to the principal points of doctrine which it raises, or which are attached to it. And, first, has the syphilitic matter invariably the same activity, the same force? I have already discussed elsewhere this grave problem, and without pretending to arrive at definite conclusions, I will signalize the following facts which observation furnishes:

1st. It is certain that the individuality of the patient plays a grand roll—this is noticed by inoculating the same primitive virus upon several persons.

2d. When there is a series of successive generations of inoculated chancres, the first give a pus sensibly more active than that of ulterior generations.

3d. If the properties of the inoculable matter are nearly exhausted in an individual syphilised, and one inoculates with this virus, an individual attacked by constitutional syphilis, but not yet submitted to treatment, or at the beginning of medication, the pustules are much more voluminous than those from which the virus is taken. If later, one employs this regenerated pus, and transplants it upon one undergoing syphilisation, it gives place to more intense manifestations. I have noticed elsewhere, that it may happen otherwise. Thus, the virulent matter, active in one person is inoculated into another, it appears to act fully at first, but after several inoculations with the same pus, the action becomes much more lively.

Dr. Clore has issued, lately, new views upon the qualities of syphilitic virus, according as the chancre is or is not indurated. The experiments which I have undertaken, upon a large scale, have not resulted precisely according to his opinion, and do not permit me to admit the absolute distinction between the simple chancre or chancroide, and the indurated chancre. It results from numerous statements, at least among us in Norway, that chancres contracted abroad, or those which are produced from them, but little time after returning to the country, are the only inoculable; at the same time that we had the inoculable chancres, we often observed suppurated buboes. The season of the year when inoculable chancres and buboes existed together, lasted from spring to the end of summer, the winter was passed, almost, always without its being possible to obtain positive results by inoculation from any kind of chancre; however, there was, during the months of winter, chancres noninoculable, which became indurated, and were followed by constitutional syphilis.

One question, still more grave, is the preservative virtue of syphilisation. How long will the immunity last? Is it equal to that given by vaccination? Is it as durable? Does it differ in its nature or effects? The answer is difficult. We cannot, I think, take artificial inoculation as a criterion, and I have not dared to undertake experiments under another form, the cure of syphilis not implying an inaptitude to contract anew the malady. The law of Mr. Ricord, that constitutional syphilis does not attack the same person twice, does not find here its application, if it is admitted that syphilisation has completely annulled, as I believe, the virus in the economy.

I have already indicated that when mercury has been administered before syphilisation, one is not sure of a guarantee against a recurrence, although it may have a very slight intensity. In these cases, if the inoculations give insufficient results, it is not rare that one obtains pustules and ulcers more extensive after the employment of iodine. In mercurialised individuals, I have repeated the inoculations after a certain lapse of time, and each time with positive effects; but I have always given iodine in the intervals.

We have in syphilisation, not only a remarkable physiological fact, but hundreds of observations exist to prove that syphilitic symptoms, exhibited in the commencement of syphilisation, disappear little by little as the inoculations are multiplied. This effect is produced more or less rapidly: the cure can only be effected after several months, but it is certain, and does not leave to the physician a moment of hesitation.

Should it be now demanded, why a practice so efficacious has not acquired more credit, the answers are numerous. I have no need to insist upon the opposition met in their origin by ideas which contradict either common feeling or received opinions; one would not have to search far to find analogous laws in the pathology of other inoculations. The most powerful objections arise from the fact, that the influence of the mercurial treatment and the obstacles it presents to syphilisation, have not been appreciated at their true value. By a happy chance of the two first patients whom I treated by this method, one had been already treated by mercury, the other, on the contrary, had not undergone any medication. In the first, accidents resisted more or less : in the other, the success was all otherwise decisive. These results led me to suppose that anterior treatment presents obstacles to the effect of syphilisation, and since then experience has transformed, for me, this hypothesis into a certitude. When mercury has not been administered, the phenomena succeed regularly ; they are continually decreasing until towards the third month, they entirely disappear. The duration of the treatment depends, besides, on that of the constitutional infection. I have already signalized the restrictions to this general rule, and recalled the case of individuals on whom are seen to reappear, during the treatment, eruptions analogous to those which existed at the moment of syphilisation. These phenomena are insignificant; they are effaced of themselves, at the end of a few weeks, and I have seen them so frequently appear and disappear that I never permit them to occupy my attention. It can be said, that the only inconvenience they present is to cause a larger sojourn of the patient in the hospital, and to give rise to the belief that syphilisation requires a longer time than it does. It would be wrong to characterize these accidents as *recidives ;* their short duration, their spontaneous cessation, show clearly that they are not really syphilitic. One comprehends that, in private practice, these *reminiscences* are of no value, the patients being reassured abstaining from all other treatment. Syphilisation appears to them much more easily supported than a mercurial course; it leaves them at liberty to attend to their affairs ; does not force them to abstain from any pleasure ; and demands no other regimen than to avoid the use of too generous wines. The syphilised has no precautions to take against atmospheric variations, and excitants of any kind do not act disadvantageously. Finally, I must proclaim again, that among individuals syphilised, and who have never previously submitted to a course of mercurial treatment, I have not observed a single case of recurrence of the disease, although many have quitted the hospital for more than three years. I pass now to the second class of the syphilised: those who have followed a mercurial treatment, with intention not to neglect any of the details which the subject, too little known, will admit of.

Certain physicians, without having against syphilisation an absolute repulsion, regard it as a last anchor of safety, when every other remedy has been exhausted—mercury, preparations of iodine, &c. Now there are doubts which exist still on the veritable anti-syphilitic powers of mercury; every one knows that mercurial treatments, the most rigorously followed, are far from always preventing a recurrence of the disease. The recidives occurs after the lapse of weeks or even years, under the form of accidents well defined and peculiar to syphilis, either as nervous troubles, hyperesthesia, paralysis or mental alienation; at other times, the patient has acquired a personal immunity; but the children who are born of him bear the too evident stigmas of syphilis, and witness that the evil has not been exhausted in its distant source.

Perhaps it will not be malapropos to mention some of the cases which I have under the eye at the time I write. The first is a man of 36 years, having suffered during fourteen years from syphilis, and submitted one after another to a series of mercurial treatments; the palatine arch is destroyed, almost every bone is the seat of exostoses, the constitution is altered to a high degree, or at least such was his state before syphilisation. Next, a man of 28 years, infected during seven years, hemiplegic several months in spite of assiduous treatment. A young girl of 21 years, who has been recently treated for and cured of a constitutional syphilis. She appeared healthy and robust, but her infant, aged six months, has been attacked by the malady, which has also reappeared in the mother. A woman of 30 years, submitted to all the severity of the method of Dzondi, and reputed perfectly cured; her first infant was still born, the second lived one day, the third eleven days, the fourth has become syphilitic at five weeks; this last has been syphilised, and will be referred to again. Finally, a woman of 41 years, not less actively treated, of whom the children have all died at the end of a few days, and who is herself hemiplegic. I could well extend this sad list, and no where is one more struck with the insufficient result of treatment, than in a little country like ours, where the patient seldom ever escapes the indefinite control of the physician. If in presence of these daily observations, repeated in spite of the care exercised, to vary the mode of employment of a remedy, we find another means exempt from the same dangers, is it wise, is it humane to prescribe it? When I represent to myself, the influence exercised by mercury, I am constrained to demand, if it would not be better to remove, entirely, the syphilisation of subjects treated by that remedy. The field for the action of therapeutic agents, is no more the same. We combat not only the disease, but syphilis allied to mercury. It seems that the virus and the remedy have submitted to a combination so intimate, that it becomes almost impossible to break it. The farther I advance, the more am I struck by the greatness of the obstacle, and the more I hesitate to emply syphilisation after the use of mercurials.

The patients who are presented in the condition of antecedent hydrargyrisation, can, besides, be divided into four categories.

1st. Those who are attacked by affections of the skin and mucous membranes yet recent. Syphilisation succeeds here, almost as well as if there had been no mercurial medication; the quantity of mercury absorbed, appears to be of no importance. On the contrary, the inveterate forms, turberculous, serpigenous and rupeacious syphilides yield less readily—either the new eruptions, more or less intense, more or less acute in their march, come in collision; or the existing eruptions are too stubborn. The inoculations give ulcers but little active: in this case, I administer iodine at the same time that I practice syphilisation, and, after under the influence of this substance, the inoculation assumes its legitimate activity. It seems that iodine enjoys the property of destroying the combination made between mercury and the syphilitic principle.

2d. Those who have suffered from affections of the bones, are not proper subjects for syphilisation, as the osseous alterations may or may not be the consequence of hydrargyric treatment.

3d. Those who are subject to nervous complaints, paralytic or hyperesthetic conditions, may experience the good effects of syphilisation. I have recounted elsewhere, a cure of syphilitic paralysis; when, on the contrary, the nervous troubles rest without their specific manifestations, the chancres are never good, the general state of health alone can be ameliorated.

Perhaps it will be asked, is it right, with such feeble chancres, to have recourse to syphilisation? I believe that, if there is no other merit attached to it, it has that of destroying the dyscrasia, and preventing an extension of the evil the physician has been unable to remove. In these cases, it is necessary to lose no time in the administration of iodine. I am far from denying the utility of iodine, but I believe it exercises upon syphilis, an action really secondary. The numerous experiments I have instituted upon this subject, have convinced me that the principal virtue of iodine, here, is as an antimercurial. It is wrong to assert that mercury cures the first secondary accidents, and iodine those which follow latter: it should be said that iodine is indicated against the accidents which appear as consequences of mercurialisation.

4th. Finally, I arrange, in the last class, those who, appearing perfectly healthy themselves, have given birth to syphilitic children. The observations I have made, are neither complete nor sufficient.

The time demanded by syphilisation, upon individuals mercurialised, is exceedingly variable: as a general rule, they require a longer medication than others; say from 4 months to a year, with mean of from six to eight months, and yet, all fear of recurrence is not removed. Of 37 syphilised patients, who had, at first, been treated mercurially, 7 had a return with symptoms of medium intensity, that which proves at least, that the inoculation had not endowed the virus with new vigor.

The third proposition which I omitted in the beginning, is relative to the general improved health which the syphilised experience. When the usual symptoms of constitutional syphilis, such as fatigue, insomnolency, rheumatic-pains, exist, they disappear with syphilisation, and afterwards the patient even gains a certain "embonpoint." Probably, physicians who have never either practiced syphilisation themselves, or seen it practiced, will receive this assertion with doubt, but experience is all in its favor: at the end of three or four weeks of treatment, the patients affirm that they feel themselves disposed, and at the end of the treatment, they are in a state to undertake the most laborious occupations. It has become almost an invariable usage to oppose to syphilisation, as a decisive argument, the danger which it entails as a consequence. It is, above all, upon the phagedenism of the first ulcers, that this is founded. It is thought reasonably enough, in appearance, that if one single chancre can produce such great disorders, a hundred inoculations ought to multiply, greatly, the peril. Facts have shown that phagedenism is a rare exception, and that artificial chancres do not provoke such accidents.

Until the present, the results I have given so briefly have been obtained from adults; and until the last months of the past year, I have only applied syphilisation to persons in the strength of age. Encouraged by the remarkable effects I witnessed, and disturbed by the too often sad consequences of the mercurial treatment, I yet hesitated to apply to infants the method of inoculation. Would infants be able to support the suppuration determined by artificial chancres? Would they not be deeply if not fatally affected? I can only say that I passed more than a year in these indecisions, but the comparative examination of treatments proposed and attempted upon infants, finished by triumphing over my scruples. It was at the end of the last year that I commenced a series of researches upon infants, undertaking these experiments with the same inquietude I experienced, when, for the first time I introduced a lancet charged with the virus, under the epidermis of an adult. The event has completely reassured me, the fears I had conceived relative to the suppuration, were vain; the artificial chancres were so small that it was with difficulty that one could borrow matter for successive inocculations.

I will content myself with resúming successively some few of the long observations I have gathered up to this date.

1st. Maren Olsdatter, $1\frac{1}{2}$ years, entered the hospital 22d September, 1855. Ulcerated mucous tubercles around the arms and labia majora, redness of the throat with alteration. The infant nursed by its mother who was syphilitic and had been treated mercurially. The first manifestations date about a month back. Olsdatter has had, in all, fifty inoculations, of which twenty-six did not succeed, leaving a total of twenty-four chancres; all the ulcerations were small, and all healed two months after the commencement of syphilisation; at the end of four months the immunity was complete, consequently requiring the same time as in adults. The syphilitic phenomena disappeared gradually to the 27th day; the last vestiges of the mucous tubercles were not effaced until the end of four months and a half. From the 9th to the 22d of February, it suffered from an attack of pneumonia, but the cure of the syphilis had taken place eighteen days before.

2d. Madeline Svensdatter, 6 months of age, entered the hospital 21st November, 1855. Mucous tubercles of different dimensions about the genital parts, one of them which is ulcerated has about the dimension of a franc piece. The infant is pale and nervous; some pustules on the head; nursed by a syphilitic woman; no indications upon the health of father or mother. This infant underwent, in all thirty-eight inocculations, of which were fifteen without result, or twenty-three artificial chancres having the same characters as in the preceding case. Six weeks after inoculation, the artificial ulcers took on a redoubled activity, at the same time that a papulous exanthema appeared. Immunity was acquired about the end of two months and twenty-two days, that is four months and a half after the commencement of syphilisation. All the symptoms had disappeared.

3d. Christensen, boy of seven weeks, entered 30th January, 1856. Observed upon the nates, internal surfaces of the thighs, the heels, and the hands, round brownish colored spots, which appear elevated above the skin, and of which the diameter is from one to two lines; many of these spots are sqamous. No affection of the mucous membranes except a light coryza. The mother was treated seven years since, by the method of Dzondi, for a constitutional syphilis. Since then, she has had four children; the first, prematurely born, did not live; the second came at eight months; the third lived eleven days; the last has been submitted to 135 inoculations,of which 104 positive. The first gave ulcers from two to three lines in diameter, and sufficiently deep; later, they cause only small pustules, often hardly furnishing inoculable matter. Immunity existed at the end of four months in spite of the considerable number of inoculations. The syphilitic accidents commenced to diminish at the fourteenth day; they disappeared at the sixth week, when a new eruption of short duration supervened. The general health is well maintained. As seen by the preceding facts, inoculations have been practiced upon infants as upon adults. I have only inoculated fewer chancres each time, and repeated the operation less frequently (four to five days of interval, sometimes beyond that time.) The development of the pustules seem slower in infants than in adults. It is not common for the pustule to become evident until the end of the second day. They attain rarely dimensions which surpass one or two lines in diameter; they are slightly characterised, softer, less neatly depressed in the centre; the redness of the areola is insignificant, often null. The consecutive alteration has the characters peculiar to syphilitic ulcers, but they are developed slowly, and tend to cover themselves with scabs under which they extend. The farther one advances with the syphilisation, the smaller and more superficial they become. The cure is not accomplished rapidly, and the matter secreted remains for a long time inoculable, (twenty-six days in the first patient.) Artificial chancres are hardly at all depressed, and but little colored by pigment.

The fears which I had conceived, were not then well grounded. The chancres, instead of being more extended, as I supposed they would be, were of small proportions. As to definitive effects, they have conformed to those given by observation on adults.

1st. There is an immunity from syphilitic virus in infants: the time is nearly the same for their cure, as for that of individuals more advanced in age, as well as that the inoculations were less numerous. The susceptility to infection of the virus, varies according to individual disposition.

2d. The existing syphilitic phenomena disappeared. The infants were exempt from all mercurial treatment. But one important circumstance should be noticed: The two first were suffering from an acquired disease; the last from hereditary syphilis. It was to this fact, that the little patient owed the long time in whch it was liable to contract the malady; it was to the mercurial treatment to which its mother had submitted, and of which it felt the effects. I have, actually, to treat two infants of six months, to whom hydrargyric preparations have been administered. The artificial ulcers on them, are more extended and more profound, but the other phenomena obey the common law.

3d. The general condition of the infants has not been altered, disadvantagiously, by the inoculations; they are as fat and healthy as possible.

I do not believe that, in comparing the results with those given by young persons treated mercurially, there is room to hesitate. The whole world knows with what difficulty infants tolerate the use of mercury, and what sad influences it exercises upon their organism when it does not cause death. The element of *time*, at this age, is not to be taken into consideration. What imports, if the little patient is cured, that it bears for a few months, true little ulcerated pustules. As for the probability of recidives,the future only can teach that which it is permitted to hope ; but should recurrences supervene, is it not at least doubtful that they will be more grave, or as grave as those which a mercurial treatment is powerless to prevent ?

